# Chemoprevention of Breast Cancer: The Paradox of Evidence *versus* Advocacy Inaction

**DOI:** 10.3390/cancers4041146

**Published:** 2012-10-29

**Authors:** Rakhshanda Layeequr Rahman, Sandhya Pruthi

**Affiliations:** 1 Division of Surgical Oncology, Texas Tech University Health Sciences Center, Amarillo Breast Center of Excellence, 1400 Coulter, Amarillo, TX 79106, USA; 2 Division of General Internal Medicine, Mayo Clinic, 200 First Street SW, Rochester, MN 55905, USA; E-Mail: Pruthi.sandhya@mayo.edu

**Keywords:** aromatase inhibitors, chemoprevention, exemestane, raloxifene, SERMs, tamoxifen

## Abstract

Women who are at high risk of breast cancer can be offered chemoprevention. Chemoprevention strategies have expanded over the past decade and include selective receptor modulator inhibitors and aromatase inhibitors. Physicians are expected to provide individualized risk assessments to identify high risk women who may be eligible for chemoprevention. It is prudent that physicians utilize a shared decision approach when counseling high risk women about their preventive options. Barriers and misperceptions however exist with patient and physician acceptance of chemoprevention and continue to impede uptake of chemoprevention as a strategy to reduce breast cancer risk. Programs to increase awareness and elucidate the barriers are critical for women to engage in cancer prevention and promote chemoprevention adherence.

## 1. Introduction

The incidence of breast cancer among high risk women is 43.4 per 1,000 at five years. This rate decreased to 22 per 1,000 high risk women who received tamoxifen in the NSABP-P1 trial [1], which was the first and largest trial in the United States to demonstrate the benefits of tamoxifen in reducing breast cancer risk. Despite research advances in breast cancer prevention with tamoxifen, the incidence of breast cancer is no longer declining [2]. In 2011 approximately 243,480 new cases of invasive breast cancer are expected to be diagnosed among U.S. women [3]. An overview of randomized breast cancer chemoprevention trials evaluating tamoxifen demonstrated a 38% risk reduction in breast cancer incidence with hormonal intervention [4]. The breast cancer chemoprevention evidence based literature and publications have undergone vigorous objective assessment by numerous organizations including American Society of Clinical Oncology [5], U.S. Preventive Services Task Force [6], American College of Obstetrics and Gynecology [7] and National Comprehensive Cancer Network [8]. The organizations consistently support the use of tamoxifen for prevention of breast cancer but advise that the discussion to initiate tamoxifen be part of an informed decision making process with emphasis on a favorable risk/benefit ratio in appropriate patients. Raloxifene, another selective estrogen receptor modulator (SERM), was approved by the Federal Drug Administration, as a chemopreventive agent for breast cancer. Determination of appropriateness for chemoprevention in general is based on a 5 year Gail Model risk score of at least 1.66% [5].

Despite endorsement by several societies and organizations, use of hormonal intervention for primary prevention of breast cancer is limited even in women whose risk/benefit ratio is in favor of preventive therapies [9,10,11,12]. Herein we present an overview of evidence regarding endocrine manipulation for prevention of breast cancer and discussion on the current status of implementation of preventive therapies in clinical practice. 

## 2. Selective Estrogen Receptors Modulators (SERM)s:

The early evidence linking endocrine manipulation to breast cancer was regression of metastatic breast cancer resulting from estrogen suppression by way of oophorectomy over a century ago [13]. This led to the evolution of endocrine therapy for treatment of breast cancer and in the last few decades the focus has been on the use of endocrine agents for prevention. Pharmacological work on tamoxifen generated the concept of selective estrogen receptor modulation based on structure-function relationship [14]. Since then additional SERMs have been have been studied as potential endocrine manipulative targets. These data are summarized below:

### 2.1. Tamoxifen

In view of its proven effectiveness against metastatic, recurrent and contralateral breast cancers, reasonable toxicity profile and positive long-term follow up, tamoxifen was the obvious first choice for assessment as a preventive therapy candidate. The earliest and most extensive studies on breast cancer chemoprevention focused on tamoxifen (Table 1) [15,16,17,18]. An overview of these studies suggests a 43% risk reduction for estrogen receptor positive breast cancer; however no effect is seen on estrogen negative cancer. The most significant finding is the continued benefit of tamoxifen on breast cancer risk reduction after completion of five-year treatment. This effect confers a further 38% risk reduction between six and ten years after initiation of therapy [19]. Tamoxifen remains the first choice for chemoprevention especially in premenopausal women who for whom the benefits outweigh the risks of adverse events. Life-threatening, tamoxifen-induced events such as thromboembolic sequelae or endometrial cancer are much higher in postmenopausal women compared to premenopausal women. It is however these life-threatening tamoxifen-induced events that continues to provide the incentive for investigators to identify new chemoprevention strategies with favorable risk/benefit ratio [1]. Understandably, women who have had a hysterectomy have a favorable risk-benefit ratio. Dose reduction to 5 mg a day (as opposed to 20 mg a day) has shown some promising results in term of risk-benefit ratio in women with an intact uterus [20,21].

**Table 1:** Studies on tamoxifen (20 mg a day) as a breast cancer preventive agent.

Study	Number of women	Median follow up (mo)	Relative Risk of ER positive cancer	Population
Royal Marsden trial [15]	2,471	158	0.6 (0.43-0.86)	Positive family history
NSABP P-1 study [16]	13,388	84	0.38 (0.28-0.5)	Gail score ≥1.66%
Italian Study [17]	5,408	132	0.77 (0.51-1.16)	Healthy post hysterectomy
IBIS-1 [18]	7,139	96	0.66 (0.50-0.87)	High risk by age and family history

### 2.2. Raloxifene

Although tamoxifen is the revolutionary SERM in breast cancer prevention, raloxifene is the first drug to be used as preventive therapy studied in the context of osteoporosis prevention [22]. It is indicated for use only in postmenopausal women. This is the first drug to be approved for two of the three indications of an ideal SERM; prevention of breast cancer and osteoporosis. However, it has not been shown to prevent coronary artery disease. Three randomized trials have assessed the efficacy of raloxifene in breast cancer prevention (Table 2) [23,24,25]. Two of these studies compared raloxifene against placebo and one against tamoxifen. The original Multiple Outcomes of Raloxifene Evaluation (MORE) study was conducted to study the impact of raloxifene on osteopenia, however, a secondary trial (Continued Outcomes of Raloxifene Evaluation—CORE study) computed breast cancer incidence during extended follow up [26]. Similarly the Raloxifene Use for The Heart (RUTH) trial investigated reduction in breast cancer and cardiovascular events among women with cardiac risk factors [24]. The largest comparative study of tamoxifen *versus* raloxifene was the Study of Tamoxifen and Raloxifene (STAR) trial and this study demonstrated that both drugs are efficacious against breast carcinogenesis, albeit raloxifene is only 78% as effective as tamoxifen. The important finding of STAR trial is the lack of proliferative effect of raloxifene on the endometrium and lower incidence of thromboembolic events compared to tamoxifen; making it an ideal strategy for postmenopausal women with a uterus.

**Table 2:** Studies on raloxifene (60 mg a day) as a breast cancer preventive agent.

Study	Number of women	Median follow up (mo)	Relative Risk of ER positive cancer	Population
CORE trial [23] *	4,011	96	0.24 (0.22-0.40)	Postmenopausal with osteoporosis
RUTH Trial [24]	10,101	67	0.45 (0.28-0.72)	At high risk of coronary events and osteopororsis
STAR [25] **	19,747	81	1.24 (1.05-1.47)	Gail score ≥1.66% and postmenopausal

* 120 mg daily also used; ** Tamoxifen *versus* raloxifene.

### 2.3. Third Generation SERMs

Continued quest to develop medications with favorable risk/benefit ratio led to the development of newer SERMs. Lasofoxifene, bazedoxifene, arzoxifene and ospemifene have been assessed in the context of osteoporosis treatment and breast cancer prevention (Table 3) [27,28]. The Postmenopausal Evaluation and Risk Reduction with Lasofoxifene (PEARL) trial specifically assessed breast cancer incidence among 8,556 postmenopausal women with low bone density randomized to 0.5 mg of lasofoxifene once a day for 5 years *versus* placebo. This trial showed a 79% risk-reduction in breast cancer, 42% reduction in vertebral fracture, 24% reduction in non-vertebral fracture 32% reduction in coronary events and 36% reduction in stroke. Lasofoxifene is currently under evaluation in the United States. The GENERATIONS trial randomized postmenopausal women to arzoxifene *versus* placebo and demonstrated a significant 70% reduction in estrogen positive breast cancer. However, there was a higher incidence of thromboembolic events and gynecologic side-effects [28].

**Table 3:** Studies on third generation SERMs as a breast cancer preventive agent.

Study	Number of women	Median follow up (mo)	Relative Risk of ER positive cancer	Population
PEARL trial [27] *	8,556	60	0.19 (0.07-0.56)	Postmenopausal with osteoporosis (59-80 year)
GENERATIONS trial [28] **	9,354	48	0.30 (0.14-0.63)	Postmenopausal with osteoporosis (>59 years)

* 0.25 mg lasofoxifene a day for 5 years; ** 20 mg arzoxifene a day for up to 5 years.

## 3. Aromatase Inhibitors (AI)s

Like the SERMs, the evidence supporting the effectiveness of aromatase inhibitors as preventive candidates stemmed from the studies on AIs as adjuvant therapy and the observed rates of decline in contralateral cancers [29,30]. These studies provided scaffolding for the design of breast cancer prevention trials assessing aromatase inhibitors in high risk women. Adjuvant studies demonstrate a rate of contralateral cancers in AI treated patients to be half of those seen in tamoxifen treated patients [31]. Since a 50% rate reduction of estrogen positive contralateral cancer is observed with tamoxifen, trials designed to investigate the effectiveness of AIs, project a 75% rate reduction. The enthusiasm in favor of AIs for breast cancer prevention stems from the fact that the adverse effects of venous thromboembolism and endometrial cancer are not seen. AIs inhibit the conversion of androgens to estrogens in adipose tissue and profoundly reduce estrogen levels in postmenopausal women [32]. This effect is not reproduced in premenopausal women due to reflex hyper-stimulation of ovaries and therefore AI’s are approved only for postmenopausal women. Three AIs are used currently in clinical practice; anastrozole and letrozole are third generation non-steroidal inhibitors and exemestane is a third generation aromatase inactivator. The studies on AIs as preventive agents are listed in Table 4.

**Table 4:** Studies on AIs as a breast cancer preventive agent.

Study	Number of women	Median follow up (mo)	Relative Risk of ER positive cancer	Population
IBIS-2 trial [32] *	6,000 accrued		Recruitment Completed-Results Pending	Postmenopausal and high risk
MAP3 trial [33] **	4,560	35	0.35 (0.18-0.70)	Postmenopausal and high risk

* 1.0 mg anastrozole a day for 5 years; ** 25 mg exemestane a day for 5 years.

### 3.1. Anastrozole

The IBIS-II is a randomized placebo controlled trial that began in 2003 (Table 4) [32]; recruitment was completed in 2012 and results are pending. The primary aim is to determine the use of anastrozole in risk reduction of invasive breast cancer in postmenopausal women. The secondary aim is directed towards the risk reduction of carcinoma *in situ*. The concern is reduced bone mineral density associated with anastrozole; therefore a substudy arm of 1,000 women has been designed to assess the impact on bone mineral density.

### 3.2. Exemestane

The National Cancer Institute of Canada Clinical Trials Group conducted MAP3 (mammary prevention) trial. The primary outcome of this large randomized placebo controlled trial was to assess the effects of exemestane on incidence of invasive breast cancer. The study enrolled 4,560 postmenopausal high risk women assigned to either exemestane (25 mg daily dose) or placebo (Table 4) [33]. Secondary end points included ductal carcinoma *in situ*, lobular carcinoma *in situ*, atypical ductal and lobular hyperplasia, hip and vertebral fracture risk, cardiac end points and quality of life measures. Exemestane reduced the relative incidence of invasive breast cancer by 65% [34]. In addition, exemestane had a beneficial effect on reducing the risk of ductal carcinoma in-situ, lobular carcinoma in-situ and atypical hyperplasia. There were no serious adverse effects related to exemestane specifically bone fractures, osteoporosis, hypercholesterolemia, and cardiovascular events. Despite the higher rate of hot flashes, insomnia, arthritis, diarrhea, and fatigue, there was no difference in self-reported quality of life between the exemestane and placebo arm.

### 3.3. Letrozole

There is currently a placebo controlled trial underway assessing breast cancer preventive capability of letrozole in BRCA mutation carriers [35]. A phase 1 dose finding trial with letrozole is also underway at the University of Arizona with a primary aim to compare the effect of lower and intermittent doses of letrozole to standard letrozole therapy on estrogen suppression and side effect profile in postmenopausal high risk women [36].

## 4. Non Endocrine Agents

None of the agents described above prevent estrogen negative breast cancer. Evidence is emerging that some agents used clinically to address other diseases are effective in reducing estrogen positive and negative breast cancers (Table 5) [37,38,39,40,41,42,43,44,45,46,47].

**Table 5:** Studies on non-endocrine agents as a breast cancer preventive agent.

Study	Number of women	Median follow up (mo)	Relative Risk of ER positive cancer	Population
Fenretinide [37]	1,739	172	0.83 (0.67-1.03)	DCIS/Stage I IDCA
Bisphosphonates				
BCNI [38]	4,039	N/A	0.38 (0.28-0.5)	Case-control/records
Chlebowski *et al.* [39]	154,768	93	0.70 (0.52-0.94)	WHI Cohort
Metformin				
Bodmer *et al.* [40]	22,621	>5-year use	0.44 (0.24-0.82)	UK Database
Bosco *et al.* [41]	4,323	>1-year use	0.81 (0.63-0.95)	Case-control 1:10
Tibolone				
LIFT [42]	4,538	34	0.32 (0.13-0.80)	Osteoporosis
Statins				
Browning and Martin [43]	~17,000	~5 years	1.01 (0.79-1.30)	7 trials overview
Bonovas *et al.* [44]	-	-	1.03 (0.93-1.14)	-
Baigent *et al.* [45]	21,575	1.09 (0.79-1.49)	Meta-analysis	-
Dale *et al.* [46]	33,776	1.33 (0.79-2.26)	Meta-analysis	-
NSAIDs				
Zhao *et al*. [47]	528,705	N/A	0.94 (0.88-1.00)	-

### 4.1. Bisphosphonates

Bisphosphonates inhibit osteoclastic activity and have been used to prevent bone resorption in metastatic disease. They have also been found to reduce bone loss induced by aromatase inhibitors [48]. Beneficial effects of bisphosphonates have been documented in terms of breast cancer recurrence [49]. Two large studies have reported breast cancer risk reduction by 30% with bisphosphonates [38,39]. Similar benefits were seen in prevention of estrogen negative breast cancer in both studies. They are generally well-tolerated, but do carry a risk of osteonecrosis of the jaw, specifically in patients on high doses of nitrogen containing compounds.

### 4.2. Adenosine Monophosphate Protein Kinase (AMPK) Activator

Metformin is the most commonly used AMPK activator in the management of type II diabetes and polycystic ovarian syndrome. Laboratory data demonstrates that this drug can also inhibit growth of breast cancer tumor cells [50]. Retrospective data on diabetics treated with metformin show a reduced incidence of breast and other cancers compared with people on other hypoglycemic agents [40,41]. The drug is associated with low toxicity and easy availability and research trials are underway in breast cancer treatment and prevention. There are ongoing studies investigating the efficacy and safety of metformin in treatment of breast cancer. Interestingly, metformin is being investigated concurrently in all three phases of clinical trials rather than the traditional sequential approach from phase I through III [51,52]. This is largely due low toxicity profile, easy availability and low cost associated with this drug along with promising data supporting its antitumor effects.

### 4.3. Statins

Inhibitors of HMG-CoA reductase have been in clinical use as lipid lowering drugs for a long time. Observational studies suggest that they may have a role in prevention of breast cancer, but four meta-analyses have found inconsistent trends with no such results [43,44,45,46].

### 4.4. Non-Steroidal Anti-Inflammatory Drugs (NSAID)s and COX-2 Inhibitors

Aspirin is an inhibitor of COX-1 and COX-2. These enzymes are expressed *in situ* and invasive breast cancer cells [53]. Epidemiological studies show about a 10% breast cancer risk reduction in aspirin users and possibly more with ibuprofen [47].

### 4.5. Other Candidates

A synthetic steroid, tibolone, showed a 68% risk reduction in breast cancer and beneficial effects on bone mineral density and cardiovascular disease postmenopausal women in the LIFT trial [42]. This finding was in contrast to the findings of Million Women study which showed an increase in breast cancer with tibolone use [54]. However, the prohibitive rate of ischemic stroke has resulted in the removal of the drug from the market.

A vitamin-A derivative, fenretinide, has been studied in the context of breast cancer prevention. Some trend was seen in premenopausal women without overall convincing evidence [37]. A study of the combination of fenretinide and tamoxifen is currently underway for premenopausal women at high risk for breast cancer [55].

New research interest is developing in evaluating and targeting the HER2 neu family for breast cancer prevention based on *in vivo* demonstration of inhibition of progression of atypia to *in situ* cancer and *in situ* to invasive cancer by lapatinib [56].

In addition, melatonin and vitamin D_3_ have been shown to down regulate growth pathways of breast cancer cells and are therefore promising for future investigation of their cytostatic effect in terms of prevention [57].

## 5. Factors Contributing to Reluctance *versus* Acceptance of Chemoprevention?

Several decades of invested time, resources and research efforts has led to major advances in chemoprevention strategies to reduce breast cancer risk specifically in high risk women. Despite this major investment, the acceptance of breast cancer chemoprevention by physicians and patients is dismal [9,10,11,12]. According to a National Health Interview Survey Cancer Control module survey of 2,000, 16% of all women residing in the United States were eligible for tamoxifen prevention by FDA criteria [7]. This accounted for over 10 million women. Five percent of these had a favorable risk/benefit index and had a 5 year Gail Model risk of at least 1.66%. This number would be 2.5 million women. Amongst all women that had a net benefit with tamoxifen, approximately 58,148 invasive breast cancers would develop over next five years and if these women received tamoxifen, 28,492 of these cancers would be prevented [58]. Notwithstanding these facts, most women do not perceive that taking tamoxifen or raloxifene will change their risk of developing breast cancer even when decision aids are provided [59]. Heisy *et al*. demonstrated that almost two-thirds of women who are at high risk of developing breast cancer, actually are interested in chemoprevention, but require more information, mainly from their primary care physicians [60]. This situation begs the question as to why are primary care and family physicians reluctant to advocate chemoprevention with their patients at high risk that might have low risk of complications with such interventions [61]. There are two major explanations for the stark discrepancy between evidence and practice of chemoprevention: (i) the difficulty and challenges with assessing individualized risk *versus* benefit associated with the use of chemoprevention; and (ii) lack of biomarkers to assess response to preventive treatment as a surrogate for actual deterrence of cancer. First, in the past few years researchers have attempted to develop tools to aid physicians with risk-benefit analysis; Gail *et al.* published an exhaustive risk-benefit analysis in 1999 [62]. This statistical model has not been widely adopted mainly because it is too cumbersome to be used efficiently in a clinic setting. More recently, Layeequr Rahman and Crawford proposed a user-friendly Chemoprevention Indication Score (CIS) to help physicians and patients in decision-making taking into account patient medical co-morbidities [63]. Freedman *et al.* have published tables assessing benefit/risk of tamoxifen *versus* raloxifene in women 50 year of age and older and in those with and without a hysterectomy [64]. Second, there have been attempts at developing phenotypic markers such as breast density. This is an important missing link in breast cancer chemoprevention debate; one cannot ignore the fact that tens of millions of people now take statins for prevention of heart disease making them the most prescribed drugs in the World. This has resulted largely from the positive bio feedback provided by the measurable low-density cholesterol levels in the blood. Currently breast density is the most powerful predictor of breast cancer in terms of attributable risk. In addition to a stark difference in risk between dense and non-dense breast tissue, it is emerging as predictor of response to preventive therapy [66]. Researchers are evaluating objective and reproducible methods of density calculation with new technological advancements, and it is hopeful that chemoprevention acceptance and uptake by patients and physicians will improve as biomarkers of response become readily available [45].

The Paradox between Evidence and Advocacy Inaction: The discussion of factors contributing to our lack of appreciation of the evidence based trials in breast cancer prevention is not complete without the mention of “advocacy inaction.” In retrospect, when the P-1 study was reported, many physicians received free Gail Model risk calculators from the pharmaceutical companies that held patents for tamoxifen. In 2000, it was estimated that 120,000 women were receiving tamoxifen as preventive treatment! This number decreased to 60,000 in 2005 [67]. Now that patents on tamoxifen and exemestane have expired, it seems that the companies are no longer interested in drug promotion for prevention. It is important to emphasize that if the intent is for risk-eligible women to receive chemoprevention counseling and be prescribed medication when appropriate, a huge promotional campaign would be required on a similar scale to what was done for implementation of statins for heart disease prevention. Physicians (and physician-based organizations) play a big role in this campaign because poor communication, inadequate education on risk benefit ratio, and concerns of toxicities leading to personal bias is a significant factor against chemoprevention usage [68]. Most national societies associated with cardiovascular disease joined forces to promote prevention and now many more people are benefiting from this preventive therapy. On the contrary, when Zeneca Pharmaceuticals launched a multimillion dollar campaign promoting tamoxifen for chemoprevention, the FDA intervened. It has been downhill since then. The updated Position Statement in June 2011 by Breast Cancer Deadline 2020 reads, “There is no current evidence that these drugs (tamoxifen, raloxifene and aromatase inhibitors) “prevent” breast cancer [69]”. The USPS taskforce recommends against chemoprevention for low or average risk women, and recommends discussion of chemoprevention in high risk women while failing to define these risk categories [70]. Whereas, both sides of risk and benefit arguments are valid, is that not true for all medical decisions we make? That’s what shared decision making and informed consent is all about. The consensus statement by Cuzick *et al.* is a step in the right direction, where the term “chemoprevention” is deemed inappropriate and to be replaced by “preventive therapy” [71]. This might remove the stigma attached with the word “chemo” which is traditionally associated with grave side-effects. Also, this group proposes the model of contralateral cancer prevention as a model for study of preventive therapies which is simpler, cheaper and more focused by design. The prevalence of breast cancer should be viewed as a public health problem with grave consequence. National and international societies committed in the field of breast health should combine efforts to promote chemoprevention counseling and endorse updates on evidence based research as appealed by Schmidt [72], to guide and support physicians towards implement of chemoprevention strategies for appropriate patients. Until we are committed to do that, it is prudent that we re-evaluate the significance and commitment to continued investment in future chemopreventive research and clinical trials.

## 6. Conclusions

Evidence-based research both at the clinical and laboratory level have resulted in significant strides in the availability of new and effective chemoprevention options to reduce breast cancer risk. Overall both SERMs and AI’s have demonstrated favorable benefit-risk balance and most likely to benefit high risk women yet these medications are underutilized by primary care providers and patients. Several significant barriers to uptake of chemoprevention have been described including inability to accurate calculate individual risk, perceived risk of serious adverse events and lack of biomarkers to determine response to medication. Given the magnitude and burden of breast cancer there is no doubt that greater emphasis needs to be made on implementation of chemoprevention medications into clinical practice. National societies with a mission to improve quality of care in breast disease must prudently assist physicians caring for high risk women by providing tools for shared decision models that encourage counseling and education about the available chemoprevention options and take into account the patient’s personal values and interest in reducing breast cancer risk.
